# Multicenter Investigation of the Initial Hemodialysis Vascular Access and Its Related Factors in Hangzhou of China

**DOI:** 10.1155/2021/6628139

**Published:** 2021-02-17

**Authors:** Qisu Ying, Yong Mao, Xiangcheng Xie, Ping Wu, Jilin Ma, Wei Zhou, Jinhua Xu, Xiao Fei, Ming Wang, Yingying Qian, Jiayu Shao, Xiaofeng Xia, Xiu Yang

**Affiliations:** ^1^Department of Nephrology, Affiliated Hangzhou First People's Hospital, Zhejiang University School of Medicine, Hangzhou, China; ^2^Department of Pharmacy, Affiliated Hangzhou First People's Hospital, Zhejiang University School of Medicine, Hangzhou, China; ^3^Department of Nephrology, The Affiliated Hospital of Hangzhou Normal University (Hangzhou Second People's Hospital), Hangzhou, China; ^4^Department of Nephrology, Hangzhou Red Cross Hospital, Hangzhou, China; ^5^Department of Nephrology, The First People's Hospital of Hangzhou Lin'an District, Hangzhou, China; ^6^Department of Nephrology, The First People's Hospital of Fuyang Hangzhou, Hangzhou, China

## Abstract

**Objective:**

To investigate the initial hemodialysis vascular access in Hangzhou and provide evidence for improving the use of autologous arteriovenous fistula by identifying factors associated with the choice of initial vascular access.

**Methods:**

We retrospectively studied the initial hemodialysis vascular access of 257 patients in five hemodialysis units in Hangzhou of China during a 21-month period (January 2018 to September 2019). A logistic regression was used to identify the risk factors of failing to use an arteriovenous fistula at the initiation of hemodialysis.

**Results:**

(1) 257 participants with mean age 67.65 ± 13.43 years old were reviewed, including 165 males (64.2%) and 92 females (35.8%). The etiologies of end-stage renal disease included diabetic nephropathy (37.35%), chronic glomerulonephritis (31.13%), hypertensive nephropathy (14.01%), and other diseases (17.51%). Only 51 patients (19.84%) received arteriovenous fistula, whereas the remaining 206 patients (80.16%) initiated dialysis with a central venous catheter. (2) Logistic regression analysis revealed that the independent risk factors for central venous catheter at the initial hemodialysis were age >70 years old (OR = 4.827, *p* < 0.01 versus ≤70 years old), chronic glomerulonephritis as the primary etiology (OR = 2.565, *p* < 0.05 versus nonchronic glomerulonephritis) and eGFR <8.5 mL/min/1.73m^2^ (OR = 2.283, *p* < 0.05 versus eGFR ≥8.5 mL/min/1.73m^2^).

**Conclusion:**

The proportion of patients using arteriovenous fistula as the initial hemodialysis vascular access in Hangzhou was still low. The choice of vascular access for the first hemodialysis was related to age, eGFR, and the primary etiology of end-stage renal disease. Increasing the proportion of planned vascular access and arteriovenous fistula at the initiation of hemodialysis is still our current goal.

## 1. Introduction

As the prevalence of chronic kidney disease (CKD) has been rising steadily, the increased incidence of end-stage renal disease (ESRD) has been recognized as a major public health problem worldwide [1, 2]. Hemodialysis (HD) continues to be the single most prevalent modality of kidney replacement therapy for patients with ESRD in China [3]. Vascular access use is an integral and important aspect of hemodialysis treatment. Two types of vascular accesses are most commonly used in hemodialysis: (1) autologous arteriovenous fistulas (AVF) that are formed from a patient's endogenous vasculature, and (2) a central venous catheter (CVC) which placed into a large vein [4]. It is reasonable to have an AVF in patients requiring HD, when consistent with their ESRD Life-Plan and overall goals of care in KDOQI Clinical Practice Guideline for Vascular Access 2019 Update [5]. AVF is viewed as being superior to CVC due to improved outcome (better patency, lower complications, and lower cost) [6]. Few multicenter researches on vascular access types at the initiation of HD have been performed previously in China. The present research investigated the type of initial HD vascular access in five hemodialysis units in Hangzhou, China, and explored the factors affecting the choice of initial vascular access, which provided evidences for increasing the use of AVF at HD initiation and improving the dialysis quality of ESRD patients.

## 2. Materials and Methods

### 2.1. Study Population and Data Sources

During a 21-month period (January 2018 to September 2019), we retrospectively studied 257 ESRD patients receiving their first hemodialysis in five HD units (Hangzhou First People's Hospital, The Affiliated Hospital of Hangzhou Normal University, Hangzhou Red Cross Hospital, The First People's Hospital of Hangzhou Lin'an District, The First People's Hospital of Fuyang Hangzhou) in Hangzhou of China.

### 2.2. Inclusion and Exclusion Criteria

Inclusion criteria: patients with ESRD (eGFR <15 mL/min/1.73 m^2^) receiving their initial HD vascular access were reviewed. Exclusion criteria: patients undergoing HD because of acute kidney injury or drug poisoning were excluded. Patients were not eligible to participate if they had at least one HD in other hospitals.

### 2.3. Methods

Heal Hemodialysis Management Information System (Heal Information Technology Company, Hangzhou, Zhejiang Province, China) was used to record patients' information, such as age, gender, eGFR at the initial HD, etiology of ESRD, and educational background. Data were analyzed to investigate the type of initial HD vascular access and to explore the factors affecting the choice of initial vascular access. Simplified MDRD formula was adopted to calculate eGFR: eGFR (mL/min/1.73m^2^) = 175 × (Scr)^−1.154^ × (age)^−0.203^ × (0.742 if female).

### 2.4. Statistical Analysis

Data were analyzed with SPSS version 19.0 (SPSS, Chicago, IL, USA). Continuous data were computed by mean ± standard deviation (SD) and were compared using an independent *t*-test. Categorical variables were compared using chi-square test. Multivariate logistic regression analysis was used to estimate the factors affecting the choice of initial vascular access. Independent variables included age, gender, eGFR at the initial HD, etiology of ESRD, and educational background. The estimated standard error of the coefficient (B) was used to establish the confidence intervals (CI) of the odds ratio (OR). *p* value less than 0.05 is statistically significant.

## 3. Results

### 3.1. Baseline Characteristics

The baseline patient demographic data are provided in Table 1. 257 ESRD participants were reviewed in this study, including 165 males (64.2%) and 92 females (35.8%), with a mean age (67.65 ± 13.43) years. The etiology of ESRD was composed of diabetic nephropathy (37.35%), chronic glomerulonephritis (31.13%), hypertensive nephropathy (14.01%), and other diseases (17.51%). The eGFR level ranged from 2 to 15 mL/min/1.73m^2^ at the start of HD with an average of (7.74 ± 3.13) mL/min/1.73m^2^. We classified patients according to their educational background: 15 cases (5.84%) with a bachelor's degree or above, 64 cases (24.90%) with secondary education, and 178 cases (69.26%) with primary education or literacy.

### 3.2. The Type of Initial Vascular Access

**Figure 1:**
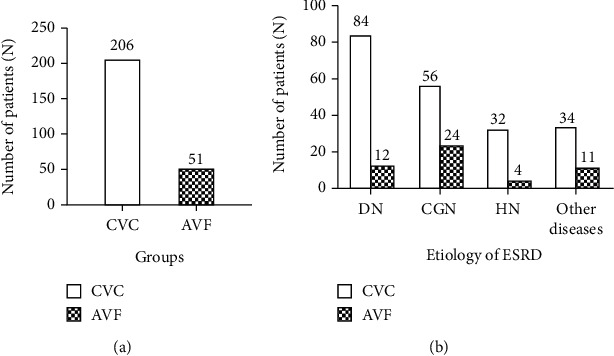
The type of initial vascular access in Hangzhou. (a) The number of patients who receive CVC/AVF. (b) The type of initial vascular access in ESRD patients with different etiology. DN: diabetic nephropathy; CGN: chronic glomerulonephritis; HN: hypertensive nephropathy.

As shown in Figure 1(a), 206 (80.16%) initiated dialysis with a CVC and the remaining 51 (19.84%) received AVF. Among the 257 ESRD patients who had received their initial HD, diabetic nephropathy (37.35%) has replaced chronic glomerulonephritis (31.13%) as the leading cause of ESRD in the 5 hemodialysis units in Hangzhou (shown in Figure 1(b)).

### 3.3. Risk Factors Affecting the Choice of Initial Vascular Access

A logistic regression model was used to estimate the independent contribution of demographic, behavioral, and medical history variables in identifying risk factors associated with the choice of initial vascular access. A forward stepwise method (using conditional likelihood for inclusion of terms) was used for model selection for both types of analysis. The ROC curve was adopted to aid in the assessment of a cutoff value used to create a dichotomous variable. The cut-off point of age was 70 years old, whereas the cut-off point of eGFR was 8.5 mL/min/1.73 m^2^. The variable with the largest positive or negative correlation with survival was considered for entry into the equation by forward selection if the level of significance obtained was less than or equal to 0.05. Once a variable was entered into the equation, the statistics for the remaining variables were used to select the next most important one for entry into the equation.

The stepwise multivariate analysis showed that independent risk factors for CVC at the initial HD were age >70 years old (OR = 4.827, *p* < 0.01 versus ≤70 years old), chronic glomerulonephritis as the primary etiology (OR = 2.565, *p* < 0.05 versus nonchronic glomerulonephritis), and eGFR<8.5 mL/min/1.73 m^2^ (OR = 2.283, *p* < 0.05 versus eGFR ≥8.5 mL/min/1.73 m2). See Table 2 for details.

## 4. Discussion

Hemodialysis continues to be the single most prevalent modality of kidney replacement therapy [7]. According to the Chinese National Renal Data System (CNRDS), nearly 340,000 hemodialysis patients were registered online at the end of 2014 [8]. Longevity to dialysis is directly proportional to the quality of dialysis, and that quality in turn depends on the reliability and integrity of the access to the patient's vascular system. This crucial connection is known as the hemodialysis vascular access. Vascular access is the “Life Line” of maintenance hemodialysis patients. The ideal hemodialysis vascular access is one that provides adequate dialysis. Complication-free access to deliver prescribed dialysis and that is also concurrently suitable for a given patient's needs. Even if there is no absolutely ideal type of hemodialysis vascular access, the Fistula First Initiative has promulgated similar recommendations. Over the past 40 years, improvements in vascular access management have enhanced patient outcomes and decreased the epidemic of access failure [9]. According to the recommendations of the Japanese guidelines [10] and KDOQI guidelines [5], experts from the Chinese Blood Purification Association recommend that AVF should be the first choice for long-term vascular access [11]. It has been suggested that the proportion of AVF as vascular access at the start of HD should be at least 85% in Renal Association Clinical Practice Guideline on Vascular Access for Hemodialysis [12]. The first edition of Chinese Hemodialysis Vascular Access Expert Consensus proposed strives to achieve more than 50% of AVF use at HD initiation in 2020 [13].

Temporary CVC, which accompanied with an increase in complications and medical costs, was independently associated with greater mortality rates compared with AVF as an initial HD vascular access [14, 15]. Several clinical guidelines related to HD vascular access suggest increasing the use of AVF at the initiation of HD [11, 12]. Although AVF are actively promoted, their use at the start of HD still failed to reach the goal worldwide. In the present study, a multivariate logistic regression analysis discovered that the risk factors for using CVC at the initial HD would include age >70 years old, chronic glomerulonephritis as the primary etiology, and eGFR <8.5 mL/min/1.73 m^2^.

Elderly patients usually suffer from a variety of underlying diseases, such as hyperlipidemia, arterial occlusion, and atherosclerosis, which may lead to an increase in the proportion of CVC use at HD initiation because of worse blood vascular conditions compared with young people [16, 17]. In the present study, it showed that patients whose age >70 years old were 4.827 folds more likely to have CVC at the initial HD compared with those whose age ≤70 years old (95% CI: 2.197-10.607, *p* = 8.9 × 10^−5^ < 0.01). There were two key local factors around age: the low acceptance rates of hemodialysis and poor vascular conditions in elderly patients. In China, the acceptance rates of hemodialysis in elderly patients are so low that some patients have to undergo emergency hemodialysis with a CVC due to severe emergency symptoms. As we have shown in the paper, among the 257 ESRD patients who had received their initial HD, diabetic nephropathy (37.35%) has replaced chronic glomerulonephritis (31.13%) as the leading cause of ESRD in Hangzhou, China. Many elderly patients with diabetic nephropathy will be accompanied by diabetic vascular disease. And their poor blood vessel conditions are not suitable for AVF.

Chronic glomerulonephritis, a known cause of CKD, often remains undiagnosed and underestimated for their insidious onset and slow progression [18]. Some patients are not diagnosed with ESRD until they have extrarenal symptoms of ESRD, such as symptoms of the digestive system, cardiovascular system, and respiratory system. Therefore, some patients with chronic glomerulonephritis as the primary etiology of ESRD are more frequently to require emergency hemodialysis, which increases the risk of starting HD with a CVC. The present results showed that patients with chronic glomerulonephritis were 2.565 folds more likely to be treated with CVC than those without chronic glomerulonephritis (95% CI: 1.144-5.753, *p* < 0.05).

With the deterioration of renal function and the decrease of glomerular filtration rate, the risk of using CVC during the first HD increases due to the rising probability of ESRD complications such as acute left heart failure, hyperkalemia, and uremic encephalopathy [19]. The logistic regression analysis showed that patients whose eGFR <8.5 mL/min/1.73 m^2^ were 2.283 folds more likely to receive CVC at HD initiation than those whose eGFR was greater than 8.5 mL/min/1.73 m^2^ (95% CI: 1.148-4.540, *p* < 0.05).

In the present study, we found that CVC use at the initial HD was still high (80.16%) among HD patients and only 19.84% received an AVF in Hangzhou. Our data were consistent with a previous report published by Zhou [20], which showed a high proportion (91.27%) of CVC usage at HD initiation in Hangzhou. Although the proportion of AVF used at HD initiation in Hangzhou was far from the 50% recommended by expert consensus [13], it rose from 8.73% to 19.84% over the period 2009-2019 as a deeper understanding of the “Fistula First” principle in nephrologists and patients.

We all know that education level affects patient compliance. Originally, we thought that educational background would affect the patients' choice of vascular access. Some patients were unable to get a good education and had little medical knowledge due to poor economic conditions. They could not accept lifelong hemodialysis, let alone have arteriovenous fistula in advance. But in the paper, we found that education background was not a risk factor that affected patients' choice of hemodialysis pathway.

Only a small percentage of patients were very well known to clinicians before they start dialysis, and most of them have chosen AVF as the initial hemodialysis vascular access in China. It was hard for clinicians to do predialysis education, because most uremic patients did not get the regularly follow-up due to poor economic conditions. In our hospital, we strengthened the predialysis education for patients, but it could not cover patients who did not follow up regularly.

We concluded that ESRD patients with age >70 years old, chronic glomerulonephritis, and eGFR <8.5 mL/min/1.73 m^2^ are more likely to receive a CVC at the initial HD. Despite increasing recommendations, the percentage of CVC use is still high and more than 60% of ESRD patients use CVC for their first HD [21, 22]. There are many factors that can lead to this condition, such as few follow-ups, ineffective patient education and counseling, late creating of AVF, immature AVF, emergency dialysis, and rapid deterioration of renal function [23, 24]. Nephrologists should strengthen the vascular access management of patients with CKD. Both doctors and patients should follow the “Fistula First” principle to reduce the unnecessary use of CVC. Increasing the proportion of planned vascular access and increasing AVF use at HD initiation is still our current goal.

## Figures and Tables

**Table 1:** Baseline characteristics of the study population at the time of ESRD onset (*n* = 257).

Parameters	CVC *n* = 206	AVF *n* = 51	*t*/*χ*^2^	*p* value
Mean age, years (SD)	69.60 (12.89)	59.78 (12.77)	4.878	<0.001 (1.89 × 10^−6^)
Male, *N* (%)	128 (62.14%)	37 (72.55%)	1.929	0.165
eGFR (mL/min/1.73m^2^) (SD)	7.56 (3.08)	8.43 (3.28)	2.11	0.086
Primary etiology of ESRD			10.767	0.013
DN, *N* (%)	84 (40.78%)	12 (23.53%)		
CGN, *N* (%)	56 (27.18%)	24 (47.06%)		
HN, *N* (%)	32 (15.53%)	4 (7.84%)		
Other diseases, *N* (%)	34 (16.50%)	11 (21.57%)		
Educational background			1.35	0.51
Bachelor's degree or above	11 (5.34%)	4 (7.84%)		
Secondary education	49 (23.79%)	15 (29.41%)		
Primary education or literacy	146 (70.87%)	32 (62.75%)		
Five hemodialysis units			5.645	0.227
A	58 (28.16%)	18 (35.29%)		
B	18 (8.74%)	9 (17.65%)		
C	13 (6.31%)	2 (3.92%)		
D	108 (52.43%)	20 (39.22%)		
E	9 (4.37%)	2 (3.92%)		

DN: diabetic nephropathy; CGN: chronic glomerulonephritis; HN: hypertensive nephropathy; A: Hangzhou First People's Hospital; B: The Affiliated Hospital of Hangzhou Normal University; C: Hangzhou Red Cross Hospital; D: The First People's Hospital of Hangzhou Lin'an District; E: The First People's Hospital of Fuyang Hangzhou.

**Table 2:** Multivariate logistic regression model for the risk factors affecting the use of CVC at the initial HD.

Independent variable (*X*)	B	SE	Wald value	*p* value	OR (95% CI)
Age	1.574	0.402	15.357	8.9 × 10^−5^	4.827 (2.197~10.607)
CGN	0.942	0.412	5.224	0.022	2.565 (1.144~5.753)
eGFR	0.826	0.351	5.543	0.019	2.283 (1.148~4.540)

**CGN**: chronic glomerulonephritis; model is adjusted for age, CGN, eGFR. Age: (age ≤70 years old) = 0; (age >70 years old) = 1; CGN: when primary disease is not CGN = 0, when primary disease is CGN = 1; eGFR:(eGFR ≥8.5 mL/min/1.73 m^2^) = 0, (eGFR <8.5 mL/min/1.73 m^2^) = 1.

## Data Availability

The data used to support the findings of this study are included within the article.
